# 20 Years of the ISCB Student Council Symposium: shaping computational biology and future leaders

**DOI:** 10.1093/bioadv/vbag097

**Published:** 2026-05-13

**Authors:** Miguel Fernandez-Martin, Ana Castillo-Orozco, Miriam Poley-Gil, Iria Pose-Lagoa, Sebastián Urquiza-Zurich, Othmane Hayoun-Mya, Diego Machado-Reyes, Mirko Treccani, Laura Veschetti, Juan D Carvajal-Agudelo, Lillian Marie Boll, Albert Garcia-Valiente, R Gonzalo Parra, Gabriel J Olguín-Orellana

**Affiliations:** Computational Biology Group, Life Sciences Department, Barcelona Supercomputing Center (BSC-CNS), Barcelona, Spain; Evolutionary Systems Biophysics Group, Life Sciences Department, Barcelona Supercomputing Center (BSC-CNS), Barcelona, Spain; Research Institute, McGill University Health Centre (RI-MUHC), Montreal, Canada; Computational Biology Group, Life Sciences Department, Barcelona Supercomputing Center (BSC-CNS), Barcelona, Spain; Evolutionary Systems Biophysics Group, Life Sciences Department, Barcelona Supercomputing Center (BSC-CNS), Barcelona, Spain; Computational Biology Group, Life Sciences Department, Barcelona Supercomputing Center (BSC-CNS), Barcelona, Spain; Advanced Center for Chronic Diseases (ACCDiS), Faculty of Chemical and Pharmaceutical Sciences, University of Chile, Santiago, Chile; Computational Biology Group, Life Sciences Department, Barcelona Supercomputing Center (BSC-CNS), Barcelona, Spain; Biomedical Engineering Department, Center for Biotechnology and Interdisciplinary Studies, Rensselaer Polytechnic Institute, New York, NY, United States; Human Nutrition Unit, Department of Food and Drug, University of Parma, Parma, Italy; Infections and Cystic Fibrosis Unit, Division of Immunology, Transplantation and Infectious Diseases, IRCCS San Raffaele Scientific Institute, Milano, Italy; Department, Vita-Salute San Raffaele University, Milano, Italy; Department of Biology, Mount Saint Vincent University, Halifax, Canada; Department, Hospital del Mar Medical Research Institute (IMIM), Barcelona, Spain; MELIS Department, Pompeu Fabra University, Barcelona, Spain; Evolutionary Systems Biophysics Group, Life Sciences Department, Barcelona Supercomputing Center (BSC-CNS), Barcelona, Spain; Departamento de Farmacología, Facultad de Ciencias Biológicas, Universidad de Concepción, Concepción, 4030000, Chile

## Abstract

Over the past two decades, the Student Council Symposium (SCS), the flagship event of the ISCB Student Council, has grown into a vital forum for early-career researchers in computational biology. Since its inception in 2005, the SCS has served as a platform for scientific exchange, skill development, and community building in a student-led, globally inclusive environment. The 20th edition, held in 2024 in Montréal, Canada, continued the symposium’s tradition of global engagement and hybrid accessibility, reaffirming a commitment to in-person dialogue. This article presents a comprehensive retrospective of the evolution of computational biology through the lens of SCS. We trace key advances from genome-scale analyses and structural modeling to single-cell and AI-driven bioinformatics. Based on SCS2024 talks and keynotes, we illustrate how emerging interdisciplinary methods have reshaped the field. We also highlight parallel efforts in global education, regional expansion, and equity, diversity, and inclusion initiatives. This retrospective shows how SCS has not only reflected the transformation of the field but also played a key role in shaping emerging leaders in bioinformatics.

## 1 Introduction

The Student Council (SC) of the International Society for Computational Biology (ISCB) was established with the mission of nurturing the next generation of computational biologists. Since its official approval by the ISCB Board of Directors in July 2004 at ISMB in Glasgow, Scotland (Gehlenborg *et al*. 2007), the ISCB-SC has provided a global platform for students and early-career researchers (ECRs) to develop their professional skills, showcase their research, and network with peers and leaders in the field. Its initiatives include organizing annual symposia, coordinating global internships, and fuelling a growing network of Regional Student Groups (RSGs) that have spanned more than 30 regions worldwide (Shome *et al*. 2016).

At the core of the activities organised by the ISCB-SC lies the Student Council Symposium (SCS), its flagship event, designed to give students and ECRs a stage to present their research and gain experience in a supportive, student-driven environment. Since its inaugural edition in 2005 in Madrid, Spain (Guigó *et al*. n.d.), the SCS has grown into a widely recognized event that has consistently provided early-career researchers with opportunities for scientific exchange, skill development, and community building. Over time, the symposium has evolved in parallel with the field of computational biology, reflecting changes in research focus while maintaining a strong emphasis on trainee-led participation.

**Figure 1:**
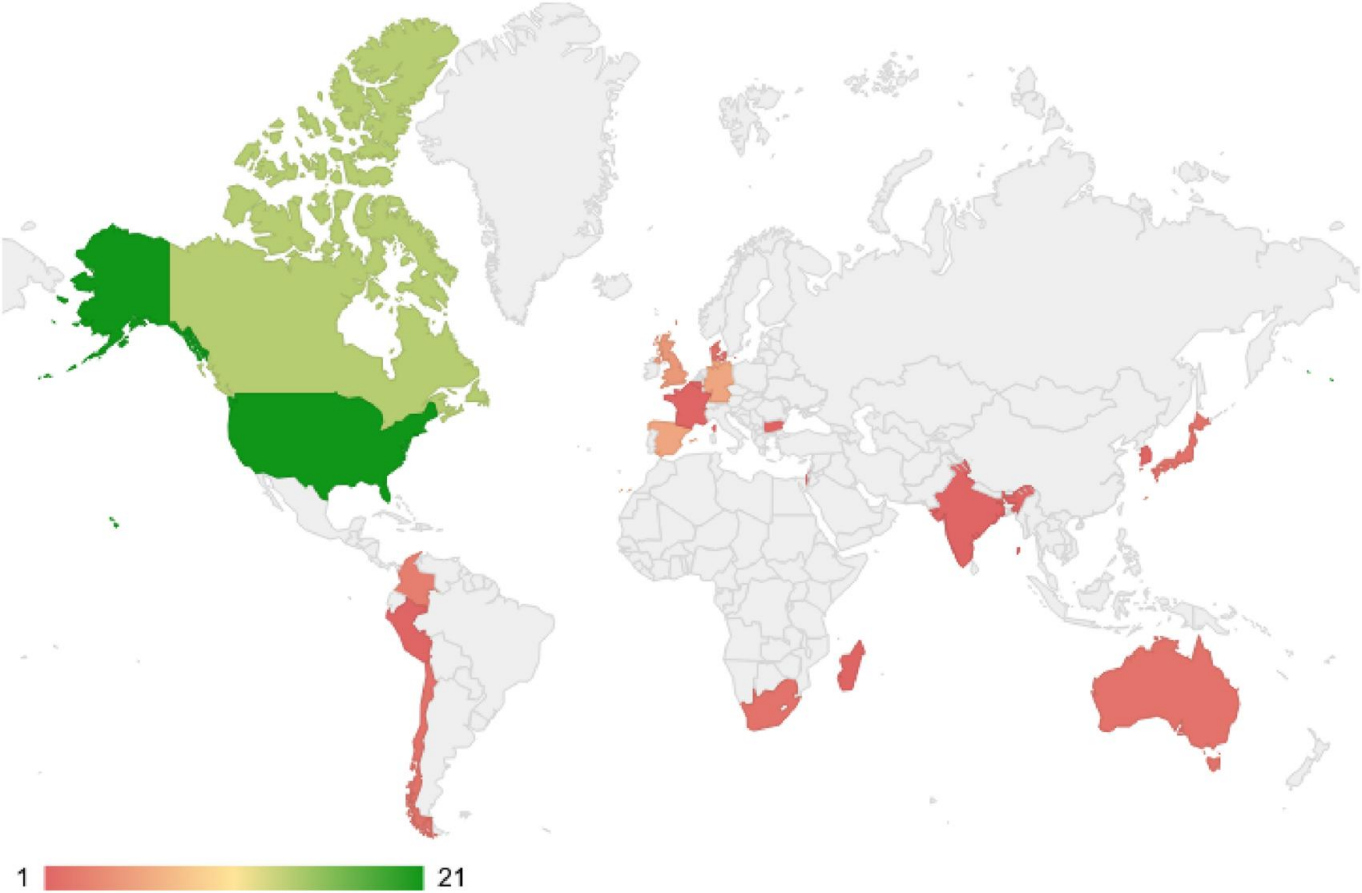
Global distribution of attendees (virtual and in-person) for SCS 2024. The color bar scale represents the number of attendees, from 1 (red) to 21 (green).

In 2024, the ISCB-SC marked a historical landmark with the 20th edition of the SCS, held in Montréal, Canada, as a satellite event of the ISCB 32nd Intelligent Systems for Molecular Biology (ISMB). Building upon the hybrid format of 19th SCS (2023), which marked a transitional phase during the post-pandemic recovery (Al Sium *et al*. 2024), SCS2024 emphasized face-to-face scientific exchange by prioritizing a return to in-person presentations and offering hybrid options for remote participants and attendees. This transition underscored the importance of live scientific collaboration and dialogue, which has historically been a defining feature of SCS editions (Gehlenborg *et al*. 2007, Peixoto *et al*. 2008, Abeel *et al*. 2009, Klijn *et al*. 2010, Grynberg *et al*. 2011, Goncearenco *et al*. 2012, Schwede and Iber 2012, Francescatto *et al*. 2015, Rahman *et al*. 2015, Cuypers *et al*. 2016, 2021, Wilkins *et al*. 2016, Bromberg *et al*. 2019, Parisi *et al*. 2019, Olguín-Orellana *et al*. 2021, Osorio-Mogollon *et al*. 2023, Al Sium *et al*. 2024, Guigó *et al*. n.d.; ‘ISCB Newsletter 9–1’ n.d.). With 72 attendees from 12 countries, the symposium reinforced its strong international presence and commitment to inclusivity (Fig. 1). The event featured keynote lectures from leading researchers in the fields of computational biology and bioinformatics, namely Dr. Manuel Corpas, Dr. Dana Pe’er and Dr. Martin Steinegger, 14 oral and poster presentations from students, and sessions focused on skill-building and career development.

**Figure 2:**
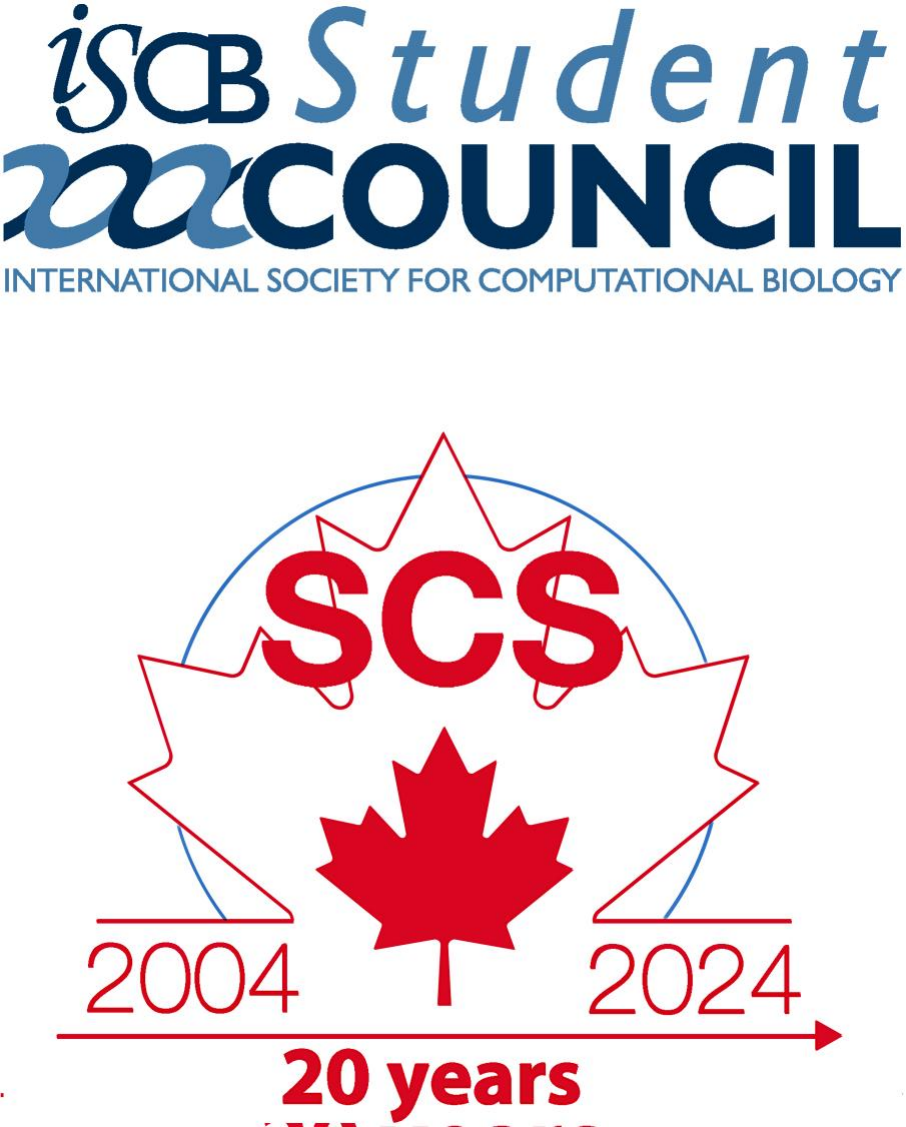
Student Council official logo (top) and special edition logo for the 20th anniversary of the Student Council Symposium celebrated in Montreal, Canada (bottom).

The organizational structure of SCS2024 played a crucial role in ensuring the symposium’s success. In particular, SCS2024 introduced an updated visual identity, including a redesigned logo and refreshed graphical elements, reflecting the symposium’s 20th-edition milestone and its evolving role within the ISCB student community (Fig. 2). Additional information about the symposium, including details on team members, schedules, and accepted abstracts, can be found in the program booklet hosted in zenodo DOI: 10.5281/zenodo.15681490

**Figure 3:**
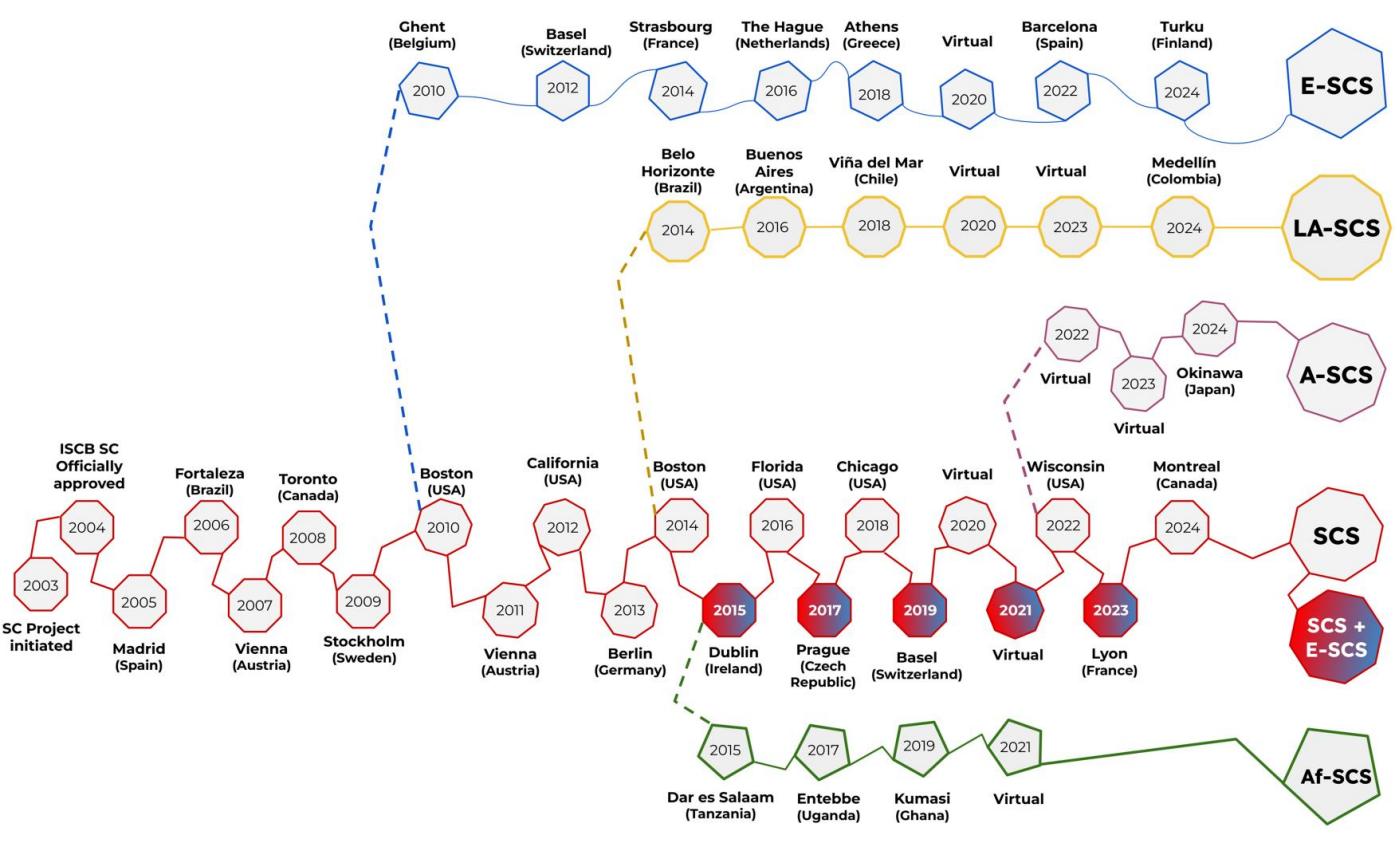
Temporal and geographic overview of ISCB Student Council Symposia (SCS) and regional Student Council Symposia.

A visual overview of all SCS editions and regional symposia, including their year and venue (city and country), is shown in Fig. 3, while Supplementary Table S1, available as supplementary data at *Bioinformatics Advances* online provides additional details such as keynote speakers and links to the published proceedings or program booklets.

## 2 From genomes and structure determination to single cells and ai-driven molecular modelling

**Figure 5:**
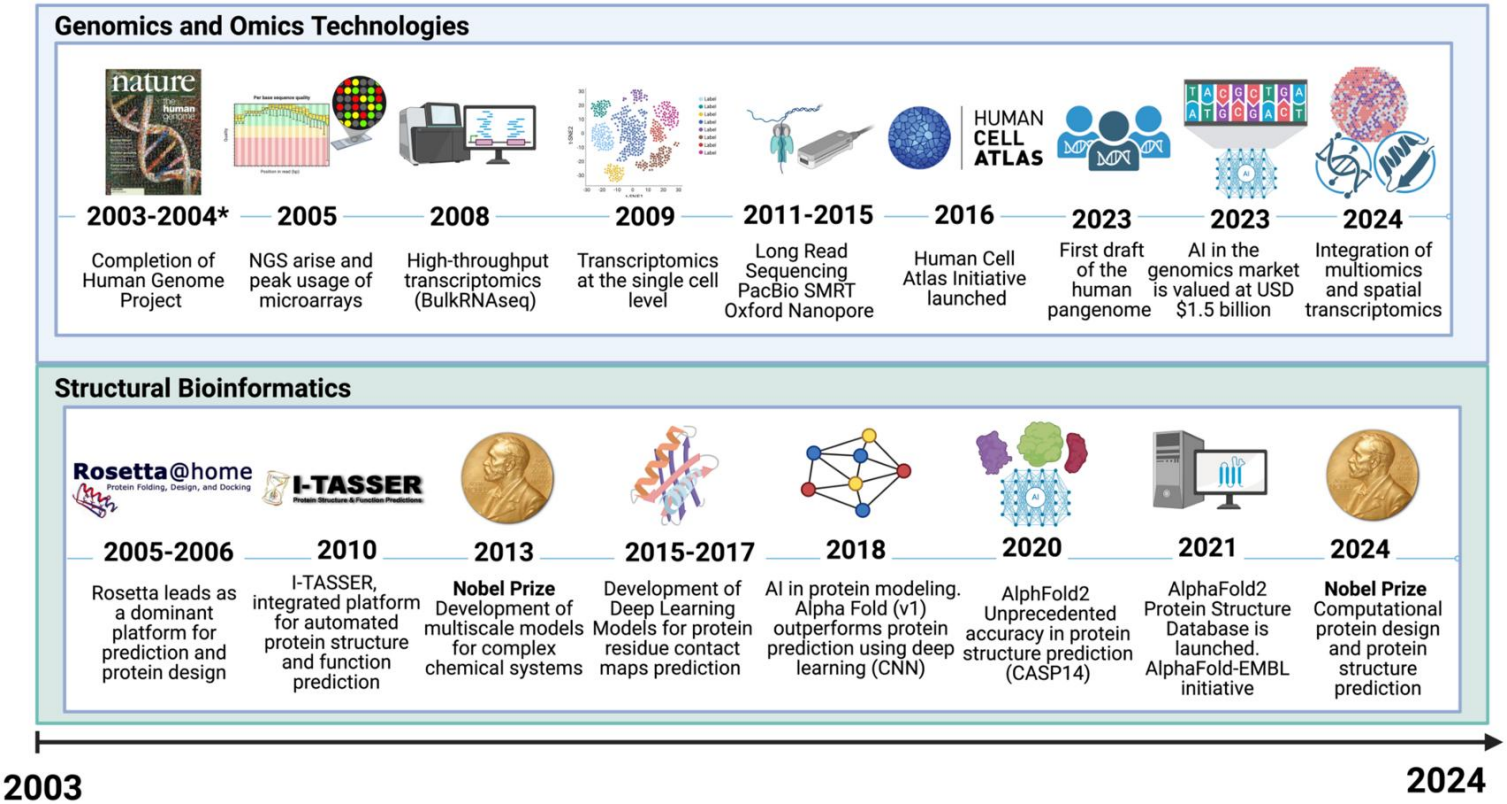
Timelines describing some of the most relevant milestones and scientific breakthroughs in Genomics/Omics Technologies and Structural Bioinformatics. *2004: Establishment of ISCB Student Council.

As the ISCB SCS marks its 20th anniversary, it provides a timely opportunity to reflect on how computational biology has transformed over the past two decades. In that time, the field witnessed a remarkable evolution in omics and structural bioinformatics, two domains that advanced in parallel and increasingly converged (Fig. 5). Early SCS meetings in the mid-2000s occurred just as high-throughput “omics” technologies revolutionized bioscience, and today’s SCS2024 highlights how far we have come, from the first genome-scale experiments to the current deep learning and AI revolution. Throughout this journey, we have spotlighted the most representative themes from SCS2024 student talks and keynotes, emphasizing how the symposium has mirrored these broader trends.

### 2.1. The early 2000s: laying the groundwork for omics and protein structures

The turn of the millennium marked the genomic revolution. The Human Genome Project delivered the first draft of the human genetic code in 2001 (Initial sequencing and analysis of the human genome 2001). In parallel, DNA microarrays introduced large-scale transcriptomic analysis as a core computational challenge. By the late 1990s, microarrays were used to classify diseases like leukemia based on gene expression patterns (Golub *et al*. 1999). The concept of “omics” rapidly expanded: researchers began talking about proteomics and metabolomics, enabled by advances in mass spectrometry (Aebersold and Mann 2003). Together, these high-throughput approaches shifted biology toward systems-level analyses, a transition reflected in the research topics presented at early SCS editions. At early SCS gatherings, students presented work on genome annotation, microarray data analysis, and protein structure prediction, cultivating upcoming research topics of the field (Gehlenborg *et al*. 2007, Al Sium *et al*. 2024, Guigó *et al*. n.d.). These beginnings firmly established that large-scale data and interdisciplinary collaboration would be the future of biology.

In parallel, structural bioinformatics emerged as a natural response to the widening gap between the number of known protein sequences and experimentally solved structures (Chou 2004). In the early 2000s, high-throughput X-ray crystallography and NMR spectroscopy provided atomic details of proteins, yet these techniques could not keep pace with explosive genome sequencing. This era saw the rise of homology (comparative) modeling tools that leveraged known structures as templates to model unknown ones (Martí-Renom *et al*. 2000). Tools such as MODELLER and SWISS-MODEL exemplified early structure prediction approaches accessible to students presenting structural bioinformatics research (Sali and Blundell 1993, Guex and Peitsch 1997). Concurrently, structural classification projects like SCOP and CATH organized proteins into evolutionary families, revealing that protein structures are more conserved than sequences (Murzin *et al*. 1995, Orengo *et al*. 1997). By the mid-2000s, these methods formed the methodological foundation of structural bioinformatics, as summarized in (Chou 2004). Around the same time, large-scale experimental efforts like the Protein Structure Initiative (PSI) contributed thousands of new protein structures, particularly for previously uncharacterized families, thus expanding the structural space available for computational modeling (National Institute of General Medical Sciences 2005).

### 2.2. The late 2000s: the next-generation sequencing boom and the rise of dynamic modeling

Mid-2000s, next-generation sequencing (NGS) technologies transformed bioinformatics. In 2005, parallel sequencers were introduced, achieving approximately 100-fold higher throughput than Sanger sequencing (Margulies *et al*. 2005). This rapid increase in throughput shifted the primary bottleneck from data generation to data interpretation. The advent of NGS gave rise to RNA-Sequencing (RNA-seq) for transcriptomics, which by 2008 had supplanted microarrays with far greater sensitivity and dynamic range (Wang *et al*. 2009). Researchers cataloged transcript isoforms and noncoding RNAs at unprecedented depth, revealing missed gene regulation complexity. In parallel, epigenomic profiling methods (e.g. ChIP-seq) enabled genome-wide interrogation of regulatory elements. For instance, the ENCODE project in 2012 integrated diverse functional genomic datasets to annotate the human genome’s noncoding “dark matter,” linking many regulatory DNA elements to gene outputs (An integrated encyclopedia of DNA elements in the human genome 2012). The scientific community learned how genetic variation across populations influences traits and disease (exemplified by the 1000 Genomes Project) (Sudmant *et al*. 2015), and began identifying molecular signatures for subclasses of diseases. These insights supported the emergence of personalized medicine as a data-driven research strategy. Pharmacogenomics, which uses genetic makeup of patients to predict drug response, became a practical reality in the late 2000s (Aneesh *et al*. 2009). This shift also democratized genomics: sequencing capacity that once required centralized genome centers became accessible to individual labs and students (Shendure and Ji 2008). This translation from genomic data to potential clinical application has been reflected in SCS presentations over time, ranging from early discussions of disease-oriented genomic analyses, such as work presented by Rasche and Herwig in SCS2007 (3rd edition) on marker identification for type 2 diabetes using integrative genomic analyses (combining quantitative trait loci with gene expression data to prioritize candidate disease genes) (Gehlenborg *et al*. 2007), to recent works in pharmacogenomics. This progression is exemplified at SCS2024 by Wisdom Akurugu’s (University of Cape Town, South Africa) on how specific DNA variants inform the choice and dosage of medications for patients in the region. Across SCS editions, we have witnessed how the Next Generation Sequencing wave has equipped students with a comprehensive “parts list” of cells (genes, transcripts, genomic variants, regulatory elements, etc) and set the stage for asking how those parts interact in health and disease.

As computing power grew, so did the ability to simulate protein dynamics. In the late 2000s and early 2010s, advances in molecular dynamics (MD) simulations enabled the exploration of protein motions on biologically relevant timescales (Shaw *et al*. 2010). Specialized hardware and improved force fields supported simulations of long-timescale conformational changes relevant to protein function (Shaw *et al*. 2008; Piana *et al*. 2012). These computational biophysical advances reinforced that protein function often depends on dynamic conformational ensembles rather than a single static structure (Karplus and Kuriyan 2005). In parallel, integrative structural biology emerged to handle systems too large or complex for any single experimental method. These modeling approaches combined data from diverse sources (e.g. X-RAY, NMR) to compute 3D models of multi-protein assemblies (Ward *et al*. 2013). Integrative modeling approaches enabled the reconstruction of large macromolecular complexes previously inaccessible to individual experimental techniques (Frank 2006, Alber *et al*. 2007, Leitner *et al*. 2016). These innovations expanded structural bioinformatics beyond single proteins, allowing the exploration of molecular machines and flexible assemblies central to cellular biology. The SCS played an early role in this arena with presentations in the late 2000s featuring nascent work on hybrid modeling of complexes, reflecting how young researchers pushed the boundaries of modeling methods at the time (Abeel *et al*. 2009). These contributions illustrate how SCS participants engaged early with emerging dynamic and integrative modeling paradigms.

### 2.3. The 2010s: systems biology, multi-omics integration, network biology, and the protein universe expansion

With genomes and transcriptomes readily in hand, the 2010s saw a shift toward systems biology: studying how genes, proteins, and metabolites interact as networks. Pioneers had outlined this vision earlier (Foundations of Systems Biology 2001), but when technology started to catch up to theory, researchers could generate multi-dimensional datasets and began to integrate them to build holistic models of cells and organisms. For example, integrating gene expression with chromatin and DNA-binding data enabled the reconstruction of gene regulatory networks (Bansal *et al*. 2007, An integrated encyclopedia of DNA elements in the human genome 2012). Multi-omics integration matured as a discipline, with strategies to connect genomic, epigenomic, transcriptomic, and proteomic information (Ritchie *et al*. 2015). Instead of examining one layer at a time, scientists now aimed to overlay all “omes” to get a panoramic view of biological systems. Crucially, these systemic approaches yielded medically relevant insights: for instance, integrating genomics with gene expression helped explain how certain non-coding variants contribute to diseases by disrupting regulatory networks (Maurano *et al*. 2012). The maturation of systems and network biology has been consistently reflected in SCS presentations over the past decade, from early student work integrating transcriptomic and translational data to identify disease-relevant regulatory mechanisms, such as Yi Zhong’s talk at SCS2014 (10th edition) which combined RNA-seq and Ribo-seq to uncover drug-sensitive genes in leukemia (Rahman *et al*. 2015), to more recent applications exemplified at SCS2024 by Sofia Rodriguez’s (Universidad San Sebastián, Chile) presentation on gene regulatory networks in rare diseases using single-cell data. Similarly, Jianlin Li’s (The University of Hong Kong, Hong Kong) talk on multi-omics and epigenetic regulation in trophoblast senescence highlighted the power of integrative analysis: combining genomic, transcriptomic, and epigenomic profiles can reveal why placental cells age prematurely. Expanding beyond human systems, Juan Picon Cossio (Universidad EAFIT, Colombia) presented the first draft genome of Talaromyces santanderensis, a cadmium-resistant fungal species, illustrating how integrated sequencing, annotation, and methylation analyses are now routinely applied to questions of environmental adaptation and bioremediation. Together, these examples reflect a broader shift during the 2010s toward systems-level bioinformatics, in which the ISCB Student Council Symposium has served as a forum for young researchers to integrate diverse data types and address biological complexity holistically.

Harnessing evolutionary sequence co-variation to infer structural contacts, at the structural level of proteins, was another transformational milestone in the 2010s. Building on earlier developments by Alfonso Valencia’s group, researchers showed that correlated mutations across protein sequences encode information about three-dimensional structural contacts (Göbel *et al*. 1994). Statistical approaches (e.g. DCA) enabled the extraction of contact information from multiple sequence alignments without requiring prior structural knowledge (Vassura *et al*. 2008). Early successes around 2011 demonstrated that these data-driven contact predictions could guide the folding of small proteins de novo (Marks *et al*. 2011). This breakthrough meant that information implicit in the growing sequence databases could be mined to predict structure. As genomic databases expanded, the accuracy of co-evolution–based modeling methods increased substantially (Ovchinnikov *et al*. 2017). By the mid-2010s, contact prediction was incorporated into protein folding pipelines, yielding moderate-accuracy models even for proteins lacking any known structural homologs (Monastyrskyy *et al*. 2016).

### 2.4. The late 2010s: the single-cell and spatial omics meets the metagenomics-led protein modeling

Perhaps the most transformative technological leap in this period was the advent of single-cell omics. Early studies around 2009–2011 demonstrated it was possible to sequence the transcriptome of an individual cell (Tang *et al*. 2009). By 2015, single-cell RNA sequencing (scRNA-seq) became high-throughput, allowing the profiling of thousands of individual cells in parallel (Jaitin *et al*. 2014). These capabilities laid the foundation for ambitious international efforts such as the Human Cell Atlas project (Regev *et al*. 2017), which aims to map every cell type in the human body as a reference for health and disease. Single-cell approaches led to the discovery of new cell subtypes (for example, previously unrecognized cell types (Macosko *et al*. 2015), and provided insights into dynamic processes like stem cell differentiation and tumor evolution. This progress was reflected at SCS2024, where Dana Pe’er’s keynote highlighted how single-cell genomics enables the identification of hidden cellular states and plasticity within tissues that are inaccessible to bulk analyses Such insights have reshaped understanding of tissue organization, disease progression, and cellular plasticity. In short, single-cell omics brought resolution to biology, fundamentally enriching our understanding of development, physiology, and disease.

In tandem, spatial transcriptomics arose as an innovative branch of omics, adding an important dimension: location. While single-cell sequencing dissociates cells from their tissue context, spatial methods enabled transcriptome profiling directly in intact tissue sections (Ståhl *et al*. 2016). These techniques made it possible to measure gene activity while preserving the coordinates of each cell in the tissue (Vickovic *et al*. 2019). This enabled new questions regarding tissue organization, cellular interactions, and spatial regulation of gene expression. Early spatial transcriptomic studies mapped, for example, the distinct gene expression neighborhoods in the brain and tumor microenvironments (Ståhl *et al*. 2016). Together, single-cell and spatial omics provided a multiscale view of tissues, linking cellular identity to tissue architecture. This shift toward cellular-resolution analysis was already reflected in SCS presentations in the late 2010s, for example through Avi Sivastrava’s work presented at SCS2019 (15th edition) on accurate gene abundance estimation from single-cell RNA-seq data (Bromberg *et al*. 2019), highlighting early engagement with cellular heterogeneity and high-resolution transcriptomic analysis. As this is still a relevant topic, at SCS2024, Ornit Nahman’s (Israel Institute of Technology, Israel) talk on spatial transcriptomics and data validity underscored the excitement and challenges in this frontier.

Ensuring data validity is critical because spatial omics are being applied to clinical samples. The integration of single-cell and spatial technologies has advanced medical research by linking cellular identity to functional tissue context. From the omics perspective, the late 2010s will be remembered as the period when cellular resolution became routine in bioinformatics (Lähnemann *et al*. 2020). The SCS mirrored this transformation: by its 16th anniversary (2020), it featured numerous presentations addressing transcriptomic, single-cell, and emerging spatial data analysis approaches (e.g., modeling mRNA abundance from regulatory sequence features) (Cuypers *et al*. 2021), indicating how quickly these methods permeated the training of young computational biologists.

In this period, another key leap came from metagenomics, which provided millions of additional protein sequences from environmental samples, which enabled co-evolutionary analyses for protein families that had previously lacked sufficient data. Martin Steinegger’s keynote at SCS2024 with his talk: “Metagenomic sequence analysis: from protein sequences to structures” emphasized how metagenomic sequences have “more than tripled the number of protein families” with enough data for accurate structure modeling. In 2017, metagenome-derived sequences were used to predict structures for hundreds of proteins without prior structural information, including many membrane proteins and novel folds, effectively solving structures in silico for more than 600 new protein families (Ovchinnikov *et al*. 2017). Metagenome-derived research has been represented at SCS since at least 2018, when keynote presentations at the Latin American Student Council Symposium discussed the use of metagenomics to identify essential genes and metabolic pathways in extreme and extraterrestrial-analog environments (Parisi *et al*. 2019). Building on this trajectory, Steinegger’s work underscored how the current expansion of the “protein universe” via environmental sequencing is allowing researchers to map out structural space far more completely than could be done with experimental structures alone. The ability to infer 3D structure from evolutionary signals transformed evolutionary biology and medicine by explaining how protein families diversify in sequence space while conserving core structural frameworks (Steinegger *et al*. 2019). This era cemented the principle that protein sequence and structure are deeply intertwined through evolution, and computational analysis of one can reveal the other.

### 2.5. The 2020s: deep learning and AI-powered bioinformatics and computational biology

The late 2010s and early 2020s witnessed an Artificial Intelligence (AI)-driven steep leap in structural bioinformatics. Traditional approaches had paired physics-based simulations with evolutionary analysis as separate paths (Jumper *et al*. 2021). Deep learning provided a way to integrate these insights and model sequence-structure relationships with unprecedented accuracy (Wang *et al*. 2017). Initial attempts applied deep neural networks to improve contact predictions (e.g., residue-residue distance maps predicted by residual neural networks), which boosted folding success rates around CASP13 (Senior *et al*. 2020). These advances culminated in late 2020 with DeepMind’s AlphaFold2, which demonstrated that protein structure prediction could routinely reach near-experimental accuracy. AlphaFold2 integrated evolutionary information with deep learning architectures capable of capturing long-range dependencies in an end-to-end manner. The result closed the accuracy gap that had persisted in protein structure prediction for decades. At CASP14, AlphaFold’s median backbone error was ∼1 Å, whereas the next-best method was approximately 2.8 Å, a stunning improvement widely celebrated in the scientific community (Jumper *et al*. 2021).

Importantly, AlphaFold2 built upon community efforts (from co-evolution to deep networks) and inspired a new wave of methods. Concurrently, related approaches, such as RoseTTAFold, further demonstrated that deep learning architectures integrating sequence, distance, and coordinate information could achieve high predictive accuracy, and the AlphaFold architecture itself was made open-source, spurring countless derivatives and applications (Baek *et al*. 2021). The AlphaFold Protein Structure Database was released in 2021 through a collaboration between DeepMind and the European Bioinformatics Institute (EMBL-EBI) and provided predicted structures for virtually any protein sequence. This resource expanded structural coverage exponentially from approximately 170,000 experimentally resolved entries in the Protein Data Bank (PDB) to over 200 million high-confidence models within just two years. The rapid impact of AlphaFold was soon reflected within the Student Council community. One of the earliest student presentations explicitly incorporating AlphaFold2 appeared at SCS2022, where Athanasios Baltzis presented work demonstrating significant improvements in protein sequence alignments using AlphaFold2-derived structural information. In the years that followed, the use of these methods expanded rapidly, culminating at SCS2024 with contributions that go beyond static structure prediction. Miriam Poley-Gil’s (Barcelona Supercomputing Center, Spain) talk exemplified this progression by applying deep neural networks to study entire protein families from a molecular biophysics perspective. Her work explores how subtle sequence variation influences stability, dynamics, and function across evolutionary variants, using approaches such as protein language models and generative models to embed sequences in latent spaces reflecting structural and biophysical constraints. The field now routinely tackles questions that were speculative twenty years ago: How might a trillion possible mutations alter the activity of an enzyme? Can proteins be designed from scratch to fold and function as desired? These questions are increasingly within reach, as illustrated by the research presented by early-career scientists at the latest editions of the ISCB Student Council Symposium.

Deep learning approaches have also transformed genomics and transcriptomics, enabling improved prediction of gene expression and functional genetic variation (Jumper *et al*. 2021). By the 2020s, the influence of this AI revolution was evident. At SCS2024, Manoj Wagle’s (The University of Sydney, Children’s Medical Research Institute, Australia) talk demonstrated a deep generative learning approach for multi-omics cell-type identification, showing how variational autoencoders and related models can integrate data from genomes, epigenomes, and transcriptomes to identify cell types across molecular layers. Samuel Davis’s (The University of Queensland, Australia) presentation on protein evolutionary modeling via machine learning provided another example: using deep models to understand and predict how proteins evolve. By training on the sequences of proteins across many species, these models (often based on protein language models or deep generative networks) learn the “rules” of protein evolution, enabling predictions of which mutations are functionally tolerated and even designing new proteins with specific traits. Prisha Rai (High Technology High School, United States) presented a novel variational autoencoder (VAE) pipeline to screen and generate candidate molecules against Tau aggregation in Alzheimer’s disease. Her approach combined generative modeling with molecular property prediction to identify candidate compounds from the ZINC database, recovering known Tau aggregation inhibitors, followed by in-vivo validation of functional effects in Tau-mutant Drosophila models. The pipeline successfully predicted Methylene Blue, a known Tau aggregation inhibitor, and validated its impact on transcriptional activity in Tau-mutant flies. Together, these studies highlight how early-career researchers are driving the convergence of multi-omics integration and AI-driven analysis, applying machine learning to accelerate drug discovery and produce experimentally verifiable insights.

More broadly, AI enables bioinformaticians to decipher the complexity inherent to omics data. That is, challenges that have traditionally proven difficult to address through manual or classical computational approaches. Deep learning methods facilitate the integration of nonlinear relationships across multi-omic features and support the discovery of novel biological signals (Zitnik *et al*. 2019). Despite these advantages, several issues regarding the interpretability of complex AI models and the necessity for rigorous biological validation remain. The bioinformatics community has responded by developing approaches to improve model interpretability and ensure that AI-derived insights are robust and biologically meaningful (Tonekaboni *et al*. 2019). This emphasis on explainability was also reflected at SCS2024, where Iria Pose-Lagoa (Barcelona Supercomputing Center and Universitat Politècnica de Catalunya, Spain) presented an integrative framework that combines gene expression data with network-based feature selection and explainable AI (XAI) techniques to address patient heterogeneity in complex diseases such as chronic obstructive pulmonary disease (COPD). Her approach, leveraging SHAP values for interpretability and clustering for patient stratification, demonstrates how modern machine learning tools can bridge biomarker discovery and personalized medicine by leveraging AI to inform clinical decision-making.

The SCS has become an important platform for showcasing such advancements, frequently highlighting early-career researchers-led initiatives that apply cutting-edge AI architectures to unresolved biological problems. Notably, the current generation of students is adopting modern machine learning techniques with the same ease that previous generations mastered clustering algorithms or PCR, signaling a profound shift in bioinformatics training paradigms.

### 2.6. Sequences, structures, evolution, and function: the biomedical impact

Over the past two decades, our theoretical understanding of the sequence-structure-function relationship has been continually refined. Early work established the tight coupling between protein structure and function, with evolutionary conservation patterns informing functional annotation (Laskowski *et al*. 2005). As more structures became available, methods for protein function annotation e.g., 3D templates for catalytic site motifs) improved, aiding genome annotation efforts).

A major milestone was the ability to map genetic variation onto protein structures, enabling the interpretation of disease-associated mutations and informing clinical variant analysis by the 2010s (Thusberg *et al*. 2011). This convergence of structural bioinformatics and genomics proved transformative for biomedical science (Chou 2004), supporting structure-guided drug discovery and the identification of therapeutically actionable vulnerabilities (Lionta *et al*. 2014). At the same time, structural modeling reshaped evolutionary biology by highlighting the functional importance of conformational flexibility, including metamorphic folds and intrinsically disordered regions, leading to a view of proteins as dynamic and evolvable entities (Wright and Dyson 1999).

Several talks at SCS2024 underscored these themes. Matthew Unger’s (University of California Santa Barbara, United States) presented work on biomolecular condensates, highlighting the functional roles of intrinsically disordered proteins (IDPs). His work focused on how low-complexity disordered regions drive liquid–liquid phase separation and the formation of membraneless organelles using computational modeling and simulations to understand how these IDPs sample myriad conformations and condense into droplets. This is a challenging problem because classical structural biology struggles with dynamic heterogeneity; however, advances in coarse-grained simulations and sequence analysis have begun to elucidate the biophysical code for phase separation (Banani *et al*. 2017). This research has broad implications for cellular organization and diseases associated with aberrant phase separation (e.g., Alzheimer’s) (Fu *et al*. 2024). Similarly, Adeline McKie’s (Queen’s University Belfast, United Kingdom) SCS2024 talk on linking structural mutations to drug discovery via synthetic lethality exemplifies the direct impact on precision medicine. Synthetic lethality (simultaneous perturbation of two genes is lethal to a cell, but either alone is tolerated) has become a promising strategy for cancer therapy, a classic example being BRCA mutations with PARP inhibitors (Previtali *et al*. 2024). McKie’s work integrates structural bioinformatics to identify how certain mutations (e.g. in tumor suppressors) create actionable vulnerabilities in cancer cells. James W. Li (Wake Forest University, USA) presented FinaleToolkit, a fast, memory-efficient computational framework for fast analysis of genome-wide fragmentation patterns in cell-free DNA. This capability opens the door to large-scale, non-invasive biomarker studies for disease diagnosis and prognosis. Structure-based approaches are now accelerating the discovery of small molecules that exploit such synthetic lethal interactions. In essence, structure-guided drug design is increasingly integrated with genetic interaction maps to identify therapeutically actionable vulnerabilities, an approach that is gaining traction in contemporary drug discovery pipelines (Previtali *et al*. 2024) and was highlighted in discussions at SCS2024.

## 3 Soft skills and culture development in computational biology over the last decades

### 3.1 Education and training in computational biology

Over the years, computational biology education has transformed from ad hoc learning paths to structured, globally informed programs. In the early 2000s, formal bioinformatics courses were relatively scarce, and most practitioners were self-taught or migrated from other disciplines (Attwood *et al*. 2015). Recognizing the need for standardized training, the ISCB convened an Education Committee in 2012 (Welch *et al*. 2012) that in 2014 published core competencies for bioinformatics curricula. This milestone report defined essential skills and knowledge areas for budding computational biologists, providing a framework to be adopted by many universities and training centers (Welch *et al*. 2014). Subsequent updates in 2016 (Welch *et al*. 2016) and 2018 (Mulder *et al*. 2018) evaluated and refined these competencies to keep pace with the fast-evolving field. This competency-driven approach helped launch specialized degree programs and guided workshops worldwide.

In parallel, community-driven and open-access training initiatives expanded rapidly. Global networks like GOBLET (Global Organisation for Bioinformatics Learning, Education and Training), established in 2012, united trainers to share curricula and materials openly (Attwood *et al*. 2015). Building upon these global efforts, regional initiatives have also strengthened bioinformatics education and capacity. The Capacity Building for Bioinformatics in Latin America (CABANA) project, launched in 2017, exemplifies such an initiative. CABANA focused on accelerating data-driven biology in Latin America through coordinated training and capacity-building activities (Campos-Sánchez *et al*. 2024) for over 800 scientists through a combination of workshops, train-the-trainer programs, research secondments, and e-learning resources. Similarly, the H3ABioNet initiative has been pivotal in developing bioinformatics expertise across Africa. Established in 2012 as part of the Human Heredity and Health in Africa (H3Africa) consortium, H3ABioNet comprises 27 nodes across 16 African countries (Mulder *et al*. 2016). The network has implemented a multifaceted training strategy, including face-to-face workshops, online courses, internships, and a train-the-trainer program. By emphasizing local trainer development, it provides a sustainable model for bioinformatics education in resource-limited settings. Additionally, the proliferation of massive open online courses (MOOCs) and online tutorials in the 2010s further democratized bioinformatics education and reinforced a culture of open, lifelong learning (Brazas and Ouellette 2013).

**Figure 4:**
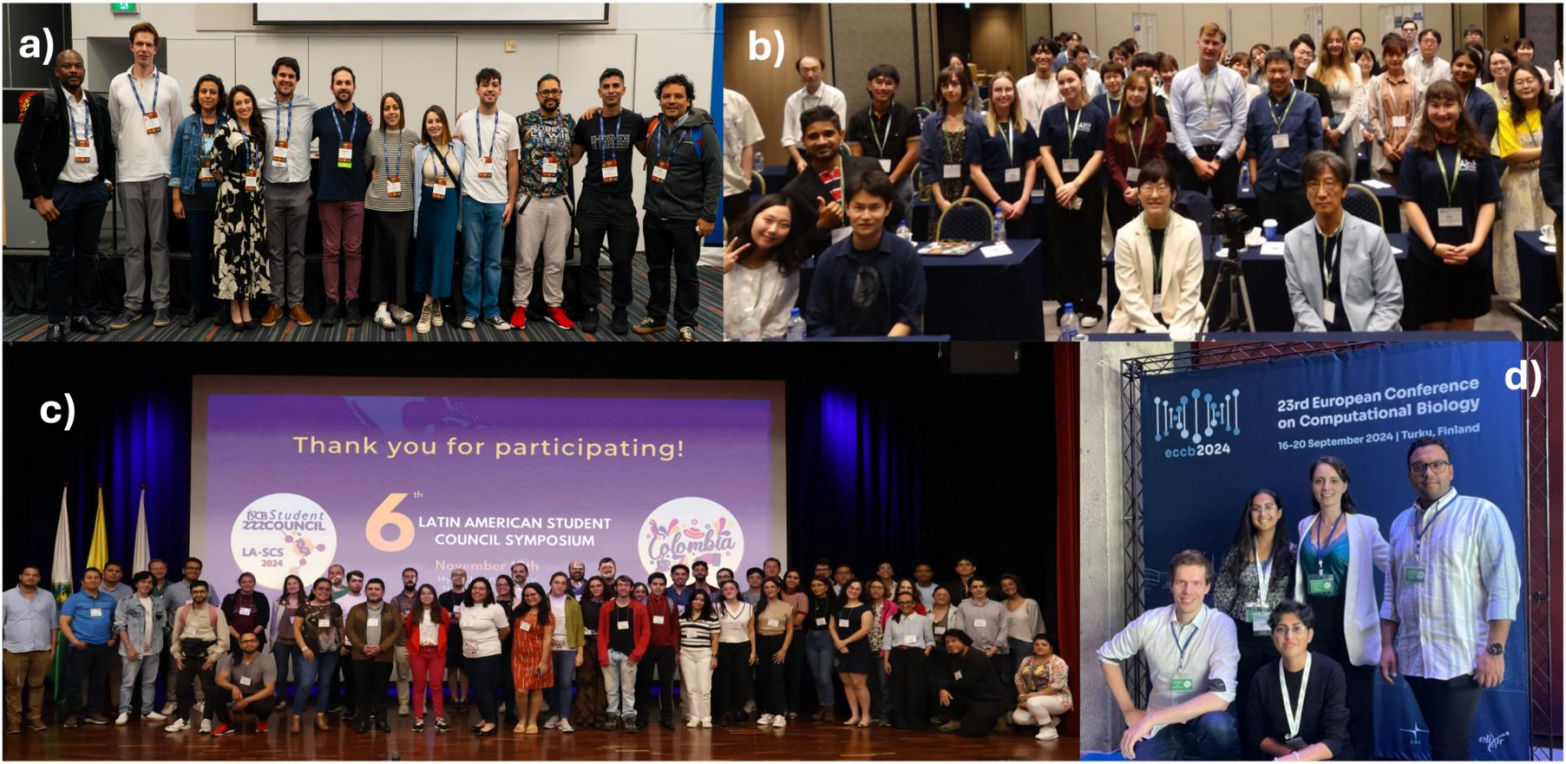
Symposia around the world in 2024. (a) SCS 2024 Organizing Team, Canada; (b) ASCS 2024 official photo, Japan; (c) LASCS 2024 Organizing Team, Colombia; (d) ESCS 2024 official photo, Finland.

Importantly, training methods have also evolved toward hands-on, experiential learning. Traditional lectures have given way to hackathons, code academies, and project-based workshops. For example, Sara Fumagalli’s (University of Milano-Bicocca, Italy) SCS2024 talk described immersive microbiome bioinformatics workshops that engage early-career researchers in real-world projects, exemplifying this shift to learning-by-doing. Such approaches reinforce not only technical skills but also soft skills like teamwork, problem-solving, and communication. The ISCB SC itself has been instrumental in this educational evolution: SCS provides young researchers a platform to present work and receive feedback in a supportive setting, cultivating presentation and networking skills that formal curricula often overlook. After 20 editions, SCS has become a training ground for the next generation, complementing academic programs by focusing on these critical soft skills (Fig. 4).

### 3.2 Global community expansion, outreach and leadership

The computational biology community has grown from a niche group to a globally connected network of researchers over the last 20 years. A key turning point was the formation of the ISCB SC in 2004, which for the first time formally organized students and young scientists in the field (Guigó *et al*. n.d.). Dr. Manuel Corpas, one of the founders and first chair of the SC, was also one of the SCS2024 keynote speakers. During his talk: “A Scientific Career Shaped by the Student Council”, he explained how he articulated his vision to “federate” students across regions, laying the roundwork for a worldwide community. The first Student Council Symposium in 2005 gathered around 100 early-career researchers, and from that seed has grown a thriving annual event. Now in 2024, the 20th SCS stands as a testament to sustained student-driven engagement, having continually connected young scientists from every continent.

One of the most significant community developments has been the creation of Regional Student Groups (RSGs) under the SC umbrella. Beginning with just four pilot RSGs in 2006 (Netherlands, India, Singapore and Korea), the network expanded to more than 30 active RSGs worldwide by the 2020s (Shome *et al*. 2019). These student-led regional communities break down geographical barriers, enabling collaboration and peer support at the local level while feeding into the global SC network. RSGs organize regional symposiums, workshops and networking events tuned to local needs and languages, greatly extending the ISCB’s outreach. For example, Europe saw the launch of the ISCB European Student Council Symposium (E-SCS) in 2010 (Moreau and Heringa 2010), Latin America followed with its first ISCB Latin American Student Council Symposium (LA-SCS) in 2014 (Parra *et al*. 2014), Africa started the ISCB African Student Council Symposium (Af-SCS) in 2015 (Souilmi *et al*. 2015), and Asia introduced its ISCB Asian Student Council Symposium (A-SCS) in 2022 (Grover *et al*. 2023), ensuring that young researchers in these regions have accessible forums to participate in the international conversation (Fig. 3).

Beyond the SC, the broader computational biology community saw parallel growth of professional societies and networks in underrepresented regions. The African Society for Bioinformatics and Computational Biology (ASBCB) and The Asia Pacific Bioinformatics Network (APBioNet) expanded their membership and training activities through the 2000s, often partnering with ISCB for conferences (Khan *et al*. 2013, Souilmi *et al*. 2015). The Open Bioinformatics Foundation (OBF) and its annual Bioinformatics Open Source Conference (BOSC) also cultivated a global following, promoting open-source practices and welcoming contributors worldwide (Harris *et al*. 2024, 202). All these efforts have helped computational biologists everywhere feel part of a global community of practice rather than isolated pockets.

Within this expanding ecosystem, the Student Council’s emphasis on peer support and inclusivity has played a formative role in early-career development. In his SCS2024 keynote, Dr. Manuel Corpas reflected on how early involvement in the SC influenced his professional trajectory, leading to collaborations and opportunities that spanned countries. His career, ranging from pioneering personal genome sequencing within his family (“Corpasome”) to coordinating international conferences, highlights the lifelong networking benefits that emerged from student community engagement. Similarly, RSG Brazil’s SCS2024 presentation on mapping Brazil’s bioinformatics training needs via community surveys showcases how regional groups today take initiative to identify gaps and drive curriculum improvements locally. By aligning their surveys with ISCB core competencies, this initiative demonstrates the interplay between global frameworks and local action. Overall, the past 20 years have seen the computational biology community become more interconnected and proactive, with the SCS serving as a yearly focal point where these connections strengthen and new initiatives emerge.

Beyond the many initiatives and activities led by the Student Council, it is equally important to highlight the broad skills that members develop by supporting this community. Over the past two decades, the Student Council has proven to be a true “incubator” for young talent, fostering the development of critical soft skills such as leadership, diplomacy, management, and communication. A retrospective look at the Council’s history shows that its previous members have steadily advanced in their careers, with many now holding leadership positions across the field such as the successful stories we learned from our SCS2024 round table (See 4. Roundtable) and the students that benefited from the ISCB Student Council Internship Program (Anupama *et al*. 2018). Notably, we have become witnesses to a new rise of a generation of leaders, such as those currently serving on the ISCB Board of Directors, who once emerged from the Student Council, thereby highlighting the importance of recognizing the Council’s role as a training ground for the future leaders of ISCB.

**Figure 6:**
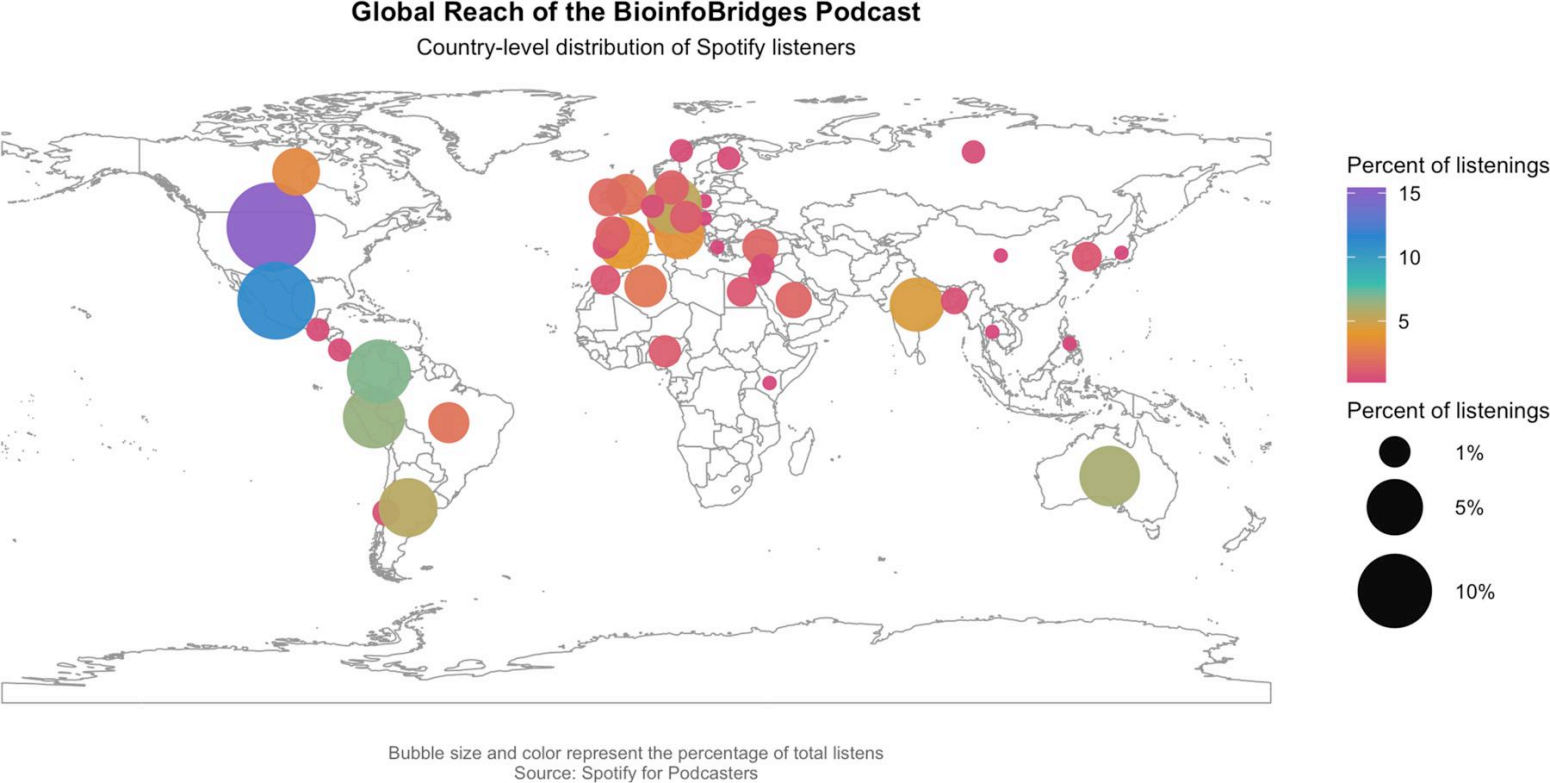
Global reach and audience demographics of the ISCB-SC Official Podcast *BioinfoBridges*. Country-level distribution of listeners based on Spotify analytics. Circles are positioned and scaled according to the percentage of total listens.

Another clear example of the skills cultivated within this environment is the creation and launch of “BioinfoBridges”, the first official podcast of the Student Council. This podcast aims to discuss a wide range of topics of great interest to young professionals and audiences interested in bioinformatics and computational biology. Episodes have explored issues related to the latest advances in the field, such as long-read sequencing technologies, network biology, machine learning, and structural bioinformatics. At the same time, the program proceeds with easy-to-understand conversations to discuss experiences from interviewed guests and provide advice to support the career journeys of its audience. Since its launch, BioinfoBridges has demonstrated substantial international reach, with listeners in 47 countries worldwide (Fig. 6). Audience engagement is strongest in North and Latin America, followed by Europe, reflecting both the geographic distribution of Student Council activities and the podcast’s language accessibility (episodes are available in English). The highest proportions of listeners originate from the United States of America (15%), followed by Latin American countries such as Mexico (11%), Colombia (7%), Peru (6%), and Australia (5%). In contrast, we have identified Asia and Africa as areas of opportunity for further growth. In response, the Student Council recognizes the importance of intentionally featuring speakers, topics, and perspectives from these regions to broaden engagement and representation across continents.

Listener demographics further indicate that BioinfoBridges successfully reaches its intended audience of trainees and early-career professionals. The age distribution is skewed toward early career stages, with the largest proportions of listeners aged 23–27 years (32.7%) and 28–34 years (30.7%), followed by 35–44 years (23.2%). Smaller fractions of the audience fall within the 18–22 year (7%) (potential undergraduate students) and 45–59 year (4.3%). The gender distribution among listeners is relatively balanced, with 50% identifying as male and 47.3% as female, though information on non-binary or LGBTQ+ identities is currently unavailable. These data suggest that the podcast effectively engages students, postdoctoral researchers, and early-career professionals across academia and industry, aligning closely with the Student Council’s mission to support the next generation of computational biologists. Although it is worth mentioning that this initiative welcomes listeners of all ages and academic stages who are interested in the field.

### 3.3 Championing equity, diversity, and inclusion

Computational biology has made concerted strides toward equity, diversity, and inclusion (EDI), recognizing that a vibrant community must reflect the diversity of its members and ideas. Two decades ago, leadership and participation in bioinformatics were skewed toward certain regions (North America, Europe) and demographics, with women and many minority groups underrepresented. This imbalance was acknowledged in community discussions; for example, a recent analysis noted that ISCB’s membership lacked gender and ethnic diversity, impacting visibility and opportunities for some groups (‘ISCB Annual Reports’, n.d.). Over the years, awareness grew that proactive measures were needed to make the field more inclusive.

In response, several EDI initiatives emerged, particularly during the 2010s. ISCB established a dedicated Equity, Diversity\& Inclusion Committee and, by 2020, released its first EDI report to transparently track diversity in conference speakers, awardees, and leadership roles. Policies such as conference Codes of Conduct and family-friendly scheduling were adopted to ensure safe, welcoming environments regardless of gender or background. Many conferences (including the ISMB) began featuring affinity group meetings (e.g. Women in Computational Biology luncheons) and honoring female and minority scientists with keynote slots and special awards. These efforts have started to pay off: the proportion of women ISCB Fellows and keynote speakers has steadily increased in recent years as the talent pool diversifies (‘September 09, 2021: ISCB Releases Inaugural Equity, Diversity, and Inclusion Report’ n.d.).

Crucially, new communities have emerged to support historically underrepresented groups. One example is the Black Women in Computational Biology Network (BWCB), founded in 2020 to connect Black female computational biologists worldwide. BWCB has since grown into an international network providing mentorship, visibility, and advocacy for black women in the field (Adams *et al*. 2022). Similar grassroots groups and events have fostered a sense of belonging and empowerment such as the first ISCB LGBTQI+ symposium as a clear example of this (Parra *et al*. 2025). Social media movements such as #LatinXBio, #WomenInBioinformatics, and #WBDS have also helped celebrate diverse researchers and build virtual communities across borders.

The ISCB and its SCS have been at the forefront of EDI progress in many ways. From its inception, the SC emphasized open participation, offering travel fellowships and outreach to students from low-income countries to attend SCS events. These travel grants (competitively awarded based on merit and need) have enabled many young scientists who could not otherwise afford it to present their work on the international stage. During 2025, a total of 17 travel fellowships were offered to students to attend to different SCS Symposia throughout the year. Such opportunities can be career-changing, helping talent from under-resourced regions gain recognition and collaborators.

**Figure 7:**
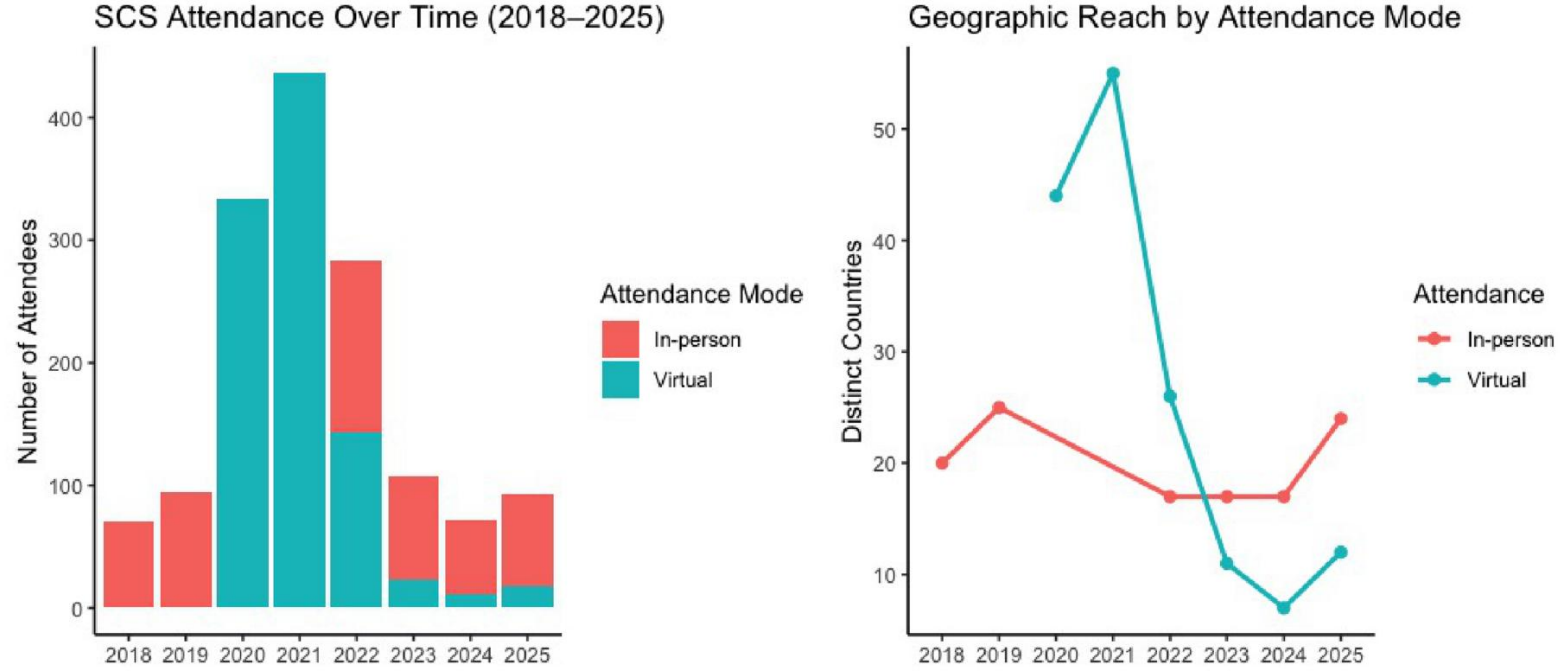
Attendance trends and geographic reach of the ISCB Student Council Symposium (2018–2025) (a) Total attendance at the ISCB Student Council Symposium (SCS) preceding the ISMB conferences from 2018 to 2025, stratified by attendance mode (in-person and virtual) (b) Geographic reach of the SCS by attendance mode, shown as the number of distinct countries represented each year.

Attendance data from 2018 to 2025 SCS events further illustrate the impact of inclusive conference formats on participation and geographic reach (Fig. 7.a). Prior to the COVID-19 pandemic, SCS attendance averaged approximately 90–100 in-person participants annually. During the fully virtual editions of 2020 and 2021, attendance increased dramatically, surpassing 400 participants each year. This increase correlated with a substantial expansion in geographic diversity, with representation from more than 50 countries worldwide, more than double that observed during most in-person editions (Fig. 7.b). Following the incorporation of hybrid formats in 2022, total attendance remained elevated relative to pre-pandemic levels, demonstrating sustained engagement from virtual participants.

While subsequent years saw a gradual decline in virtual attendance and a recovery for in-person participation to levels comparable to 2018–2019, the number of distinct countries plateaued between 2022 and 2024. A modest improvement was observed in 2025, suggesting a gradual rebound in international travel participation. This highlights the notion that while in-person conferences remain central to community building, virtual and hybrid formats substantially lower barriers to participation and enhance global inclusivity. Accordingly, we recognize the importance of continuing efforts to ensure that upcoming conferences feature virtual and hybrid events to advance our mission of making bioinformatics accessible to everyone.

Beyond conference formats, the SC’s RSGs inherently promote inclusion by decentralizing activities. For instance, RSG committees in Africa and Latin America often operate in multiple languages and tailor events to the local cultural context, lowering barriers to entry. The SC’s own leadership has grown more geographically diverse; by the 2020s, Chairs and officers have hailed from every inhabited continent, creating role models for early-career researchers worldwide (Hassan *et al*. 2018). While challenges like black and Indigenous populations are still markedly underrepresented in computational biology Ph.D. programs (Adams *et al*. 2022) remain, the community’s commitment to EDI is stronger than ever. The celebratory tone of SCS2024 reflected this ongoing commitment, emphasizing how inclusion enriches computational biology through broader perspectives and participation.

## 4 Roundtable: the student council’s impact on researchers’ personal and scientific careers

### 4.1. Topic and speaker selection

**Figure 8:**
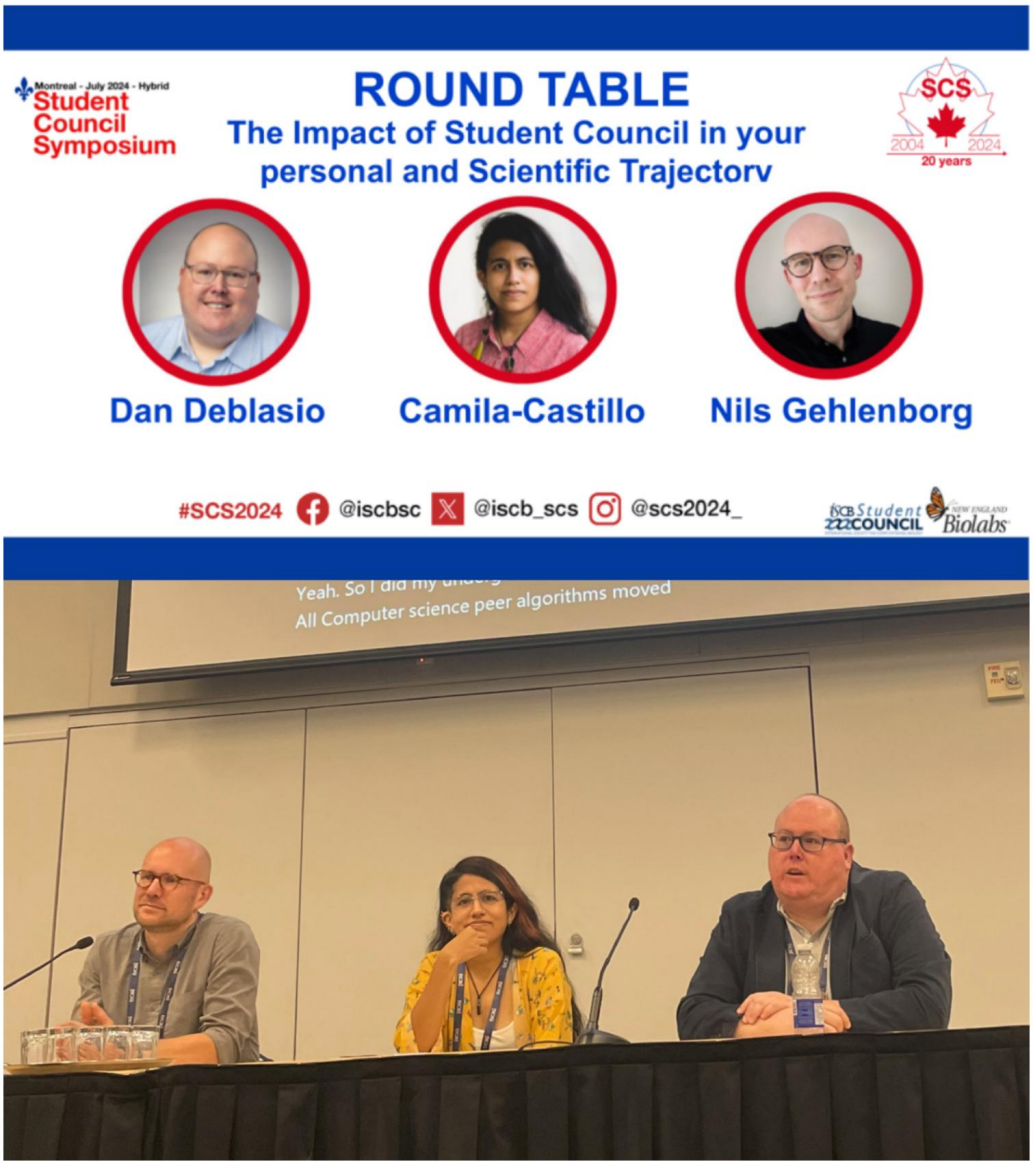
Designed posts to announce the final round table speakers (top) and a live picture during the event (bottom).

In the context of the 20th anniversary of the SCS, the program committee organized a roundtable discussion focused on the SC’s impact on personal and academic development. The topic, “The impact of the SC on your personal and scientific career” guided the selection of participants with active involvement in the ISCB SC and related activities. Three panellists committed to attending the session: Dan DeBlasio, Camila Castillo Vilcahuaman and Nils Gehlenborg (Fig. 8).

Dan DeBlasio is an Assistant Teaching Professor in the Computational Biology Department at Carnegie Mellon University, United States. From January 2017 to January 2019, he served as the SC Representative on the Board of Directors of the ISCB, contributing to the advancement of student engagement within this society (DeBlasio n.d.). Camila Castillo is the founding president of the ISCB RSG Peru and a researcher at the Microbial Ecophysiology Lab at Fundación Ciencia y Vida, Chile. In addition to her research, she is actively involved in science communication and education, leading workshops and promoting scientific literacy throughout Latin America (‘Directorio de Recursos Humanos afines a la CTI’ n.d.). Nils Gehlenborg is an Associate Professor of Biomedical Informatics and the Director of the Master of Biomedical Informatics program at Harvard Medical School, United States. He served as the Chair of the Student Council from 2010 to 2011, before being elected as the SC Representative on the ISCB Board of Directors (‘Nils Gehlenborg’ n.d.). Fun fact: Nils designed the SC logo that is still in use today.

The round table featured a structured one-hour conversation, beginning with brief introductions, followed by a moderated Q\&A session and an open floor for audience questions.

The experiences shared during the roundtable illustrated the broader professional impact of Student Council involvement beyond traditional academic activities. A key advantage of SC involvement is the opportunity to develop skills not typically covered in academic training, such as the importance of salesmanship and event logistics, which are crucial skills for any scientist looking to navigate funding landscapes and promote their research effectively. Another critical advantage of SC participation is the establishment of a strong professional network. Panelists noted that early engagement often led to mentorship opportunities, collaborations, and later leadership roles within ISCB. Camila, in particular, underscored how these networks have been instrumental in reinforcing bioinformatics communities in underrepresented regions, such as Latin America. This is a particularly good example of how SC participation provides opportunities to shape the bioinformatics community and help mentor the next generation of scientists. Each panelist’s career path demonstrates how SC engagement is not merely a complementary experience but a catalyst for long-term scientific success. Whether through developing essential soft skills, forming global research networks, or stepping into leadership roles, their experiences highlight the importance of student-led scientific organizations in career development.

A number of professional societies operate formal student councils or student-centered committees. For instance, the American Statistical Association (ASA) (‘Meet the Student Council’ n.d.)supports a Student Council made up of representatives from its technical committees who organize student events such as orientations and receptions. The Society of Physics Students (SPS) (‘SPS’ n.d.) includes student zone representatives in its National Council and Executive Committee, giving students a formal role in society governance. Similarly, the Society of Economic Geologists (SEG) (‘The Society of Economic Geologists (SEG)’ n.d.) runs a Students Committee to advise on student concerns and programming. In comparison, ISCB’s Student Council stands out both in its global reach, combining a long-running annual symposium with a global network of RSGs, which together provide periodical international leadership and training opportunities for early-career researchers.

A transcript of the questions and the responses from the panelists are collected in the program booklet, available in zenodo DOI: 10.5281/zenodo.15681490

## 5 Future perspectives

In the upcoming year the ISCB Student Council aims to build upon its existing outreach, education, and community-building initiatives by strengthening strategic partnerships, expanding global accessibility, and improving inclusivity across regions. Recent collaborations with ISMB-associated initiatives, such as contributions to social media and outreach activities for the HitSeq track and the Canadian Bioinformatics Hub Conference, illustrate the Student Council’s growing role in amplifying educational content and engaging early-career audiences within the broader ISCB ecosystem.

A key component of these future efforts is the continued collaboration with the Global Bioinformatics Education Summit (GBES). GBES is an annual initiative that brings together educators, professionals, entrepreneurs, and other stakeholders to develop strategies to make bioinformatics education more accessible and equitable worldwide. Through a successful edition (GBES 2025 Mexico City), organized in partnership with the Student Council, this collaboration has demonstrated the value of integrating student perspectives into discussions on educational frameworks and capacity-building.

Building on these experiences, the Student Council plans to expand and formalize similar activities to further expand the educational and professional development ecosystem surrounding ISMB and ISCB events.

In parallel, attendance data across our SCS symposia highlight the importance of virtual participation as a mechanism to increase visibility, reduce barriers to access, and foster broader international engagement. Enhancing hybrid and online components will therefore remain a priority to ensure more inclusive participation across geographic and economic contexts.

Finally, while current engagement data indicate strong participation from North America and Europe, the Student Council recognizes a clear opportunity to expand its presence in underrepresented regions, particularly Asia and Africa. Future outreach efforts will prioritize regional representation by featuring speakers, educators, and community leaders from these regions in podcasts, seminars, and other student council activities. This approach is intended to increase global connectivity and support the continued growth of an inclusive, worldwide computational biology community.

## 6 Conclusion

The 20^th^ edition of the ISCB Student Council Symposium 2024 (SCS 2024) represents an important moment to reflect on the trajectory of the symposium and the broader development of computational biology over the past two decades. Since the creation of SCS in 2004, bioinformatics has steadily grown from a supporting discipline into a core component of the life sciences, accompanying advances that have shaped modern biological research. Some of these advances, including developments in computational chemistry, cryo-electron microscopy, and protein structure prediction, have been widely recognized by the scientific community. In parallel, SCS has transformed from a small, student-organized event into a globally recognized forum that showcases emerging research and provides a space for early discussions on future directions in the field. From its early days, featuring student-led contributions on gene expression clustering, genome annotation, and comparative modeling, SCS has grown in parallel with advances in genomics, structural bioinformatics, systems biology, and artificial intelligence.

As new technologies emerged, SCS presentations expanded to include integrative omics, network-based analysis, and increasingly data-driven approaches to biological questions. Advances in structural bioinformatics were similarly reflected within the symposium, progressing from early structure-function analyses to more sophisticated modeling strategies. By the 2010s, as single-cell technologies, multi-omics integration, and co-evolutionary methods became widespread, SCS increasingly served as a forum where early-career researchers engaged with these emerging technologies. Contributions spanning protein modeling, regulatory genomics, and systems-level analyses illustrated how rapidly new approaches were incorporated into student research.

In the early 2020s, artificial intelligence became a unifying framework across the life sciences. At SCS, this transition was evident through presentations on deep learning for protein modeling, generative methods for single-cell analysis, and explainable AI approaches for biomedical applications. Together, these contributions highlight the role of the symposium as a space where early-career researchers explore, discuss, and critically assess emerging computational methods as they gain traction within the broader field.

Beyond technical advances, the symposium has played a vital role in shaping the culture of computational biology. Education has grown more structured and inclusive, blending core competencies with experiential learning. Within this context, SCS has supported hands-on training, leadership development, and open scientific exchange among early-career researchers. The expansion of RSGs and the Council’s sustained focus on equity, diversity, and inclusion have reinforced the symposia role as a community-oriented forum, where mentorship, representation, and collaboration complement scientific exchange.

Over the past two decades, many former Student Council members have progressed into leadership roles across academia, industry, and professional societies, including ISCB. Their trajectories highlights how early involvement in the Student Council can contribute to sustained professional development and continued service to the society. Looking ahead, the ISCB-SC is well positioned to continue shaping the future of computational biology. Its commitment to empowering young researchers, fostering interdisciplinary dialogue, and expanding global access to scientific participation ensures that SCS will remain a vital force in both scientific progress and community growth. As the field continues to evolve alongside rapid technological change, the legacy of SCS will endure, not only in the breakthroughs it has helped incubate, but in the diverse, connected, and inspired generation of scientists it continues to support.

## Supplementary Material

vbag097_Supplementary_Data

## Data Availability

No data was generated in the preparation of this manuscript.
