# Supplementary material for: 20 Years of the ISCB Student Council Symposium: shaping computational biology and future leaders
Source: Bioinform Adv. 2026 May 13;6(1):vbag097. doi: 10.1093/bioadv/vbag097 (PMC13171220; doi:10.1093/bioadv/vbag097)
Supplement: vbag097_Supplementary_Data [file vbag097_supplementary_data.pdf]

Supplementary Table 1. Previous editions of the Student Council Symposium.

Edition	Year	City	Country	Host Conference	Keynotes	Source
1st SCS	2005	Madrid	Spain	ECCB 2005	Not reported in online summary	https://drupal.iscbsc.org/scs-previous-symposiums#scs2005
2nd SCS	2006	Fortaleza	Brazil	ISMB 2006	Dr. Phil Bourne; Dr. Reinhard Schneider; Dr. Julio Collado-Vides	https://drupal.iscbsc.org/scs-previous-symposiums#scs2006
3rd SCS	2007	Vienna	Austria	ISMB/ECCB 2007	Dr. Janet Thornton; Dr. Anna Tramontano; panelists included Dr. Rita Casadio, Dr. Tan Tin Wee	https://bmcbioinformatics.biomedcentral.com/articles/10.1186/1471-2105-8-S8-I1
4th SCS	2008	Toronto	Canada	ISMB 2008	Dr. Burkhard Rost; Dr. Mark Gerstein; Dr. Timothy Hughes	https://bmcbioinformatics.biomedcentral.com/articles/10.1186/1471-2105-9-S10-I1
5th SCS	2009	Stockholm	Sweden	ISMB/ECCB 2009	Not fully listed on summary (meeting report exists)	https://drupal.iscbsc.org/scs-previous-symposiums#scs2009
6th SCS	2010	Boston	USA	ISMB 2010	Dr. Gary Bader; Dr. David Altshuler; Dr. Larry Hunter	https://drupal.iscbsc.org/scs-previous-symposiums#scs2010
7th SCS	2011	Vienna	Austria	ISMB/ECCB 2011	Dr. Chad Myers; Dr.	https://drupal.iscbsc.or

					Ivet Bahar; Dr. Curtis Huttenhower	g/scs-previous-symposiums#scs2011
8th SCS	2012	Long Beach	USA	ISMB 2012	Dr. Robin Dowell, Dr. Matthew Hibbs, and Dr. Jonathan Eisen	https://drupal.iscbsc.org/scs-previous-symposiums#scs2012
9th SCS	2013	Berlin	Germany	ISMB/ECCB 2013	Dr. Alex Bateman; Dr. Satoru Miyano; Dr. Gonçalo Abecasis; and Dr. Cheng Soon Ong (short presentations)	https://drupal.iscbsc.org/scs-previous-symposiums#scs2013
10th SCS	2014	Boston	USA	ISMB 2014	Dr. David Bartel; Dr. Ashelee Earl	https://f1000research.com/articles/4-154
11th SCS	2015	Dublin	Ireland	ISMB/ECCB 2015	Dr. Des Higgins; Dr. Ruth Nussinov	https://drupal.iscbsc.org/scs-previous-symposiums#scs2015
12th SCS	2016	Orlando	USA	ISMB 2016	Dr. John Quackenbush; Dr. Janet Thornton	https://drupal.iscbsc.org/scs-previous-symposiums#scs2016
13th SCS	2017	Prague	Czech Republic	ISMB/ECCB 2017	Dr. Christine Orengo; Dr. Johannes Söding	https://drupal.iscbsc.org/scs-previous-symposiums#scs2017
14th SCS	2018	Chicago	USA	ISMB 2018	Dr. Lucia Peixoto; Dr. Philip Bourne	https://drupal.iscbsc.org/scs-previous-sympo

						siums#scs2018
15th SCS	2019	Basel	Switzerland	ISMB/ECCB 2019	Dr. Barbara Treutlein; Dr. Christophe Dessimoz; Dr. Fabian Birzele	https://drupal.iscbsc.org/scs-previous-symposiums#scs2019
16th SCS	2020	Virtual	Global	ECCB 2020	Professor Elana J. Fertig; Professor Hamed S. Najafabadi	https://f1000research.com/articles/10-443/v1
17th SCS	2021	Virtual	Global	ISMB 2021	Prof. Wolfgang Huber; Prof. Nicola Mulder; Prof. Yana Bromberg	https://pubmed.ncbi.nlm.nih.gov/36704314/;
18th SCS	2022	Madison	USA	ISMB 2022	Dr. Marinka Zitnik; Dr. Janet Thornton	https://scs2022.iscbsc.org/.
19th SCS	2023	Lyon	France	ISMB/ECCB 2023	Prof. Anaïs Baudot; Prof. Burkhard Rost	https://academic.oup.com/bioinformaticsadvances/article/4/1/vbae028/7639973.
20th SCS	2024	Montreal	Canada	ISMB 2024	Dr. Dana Pe'er; Dr. Manuel Corpas; Dr. Martin Steinegger	https://iscbsc.org/scs2024/.
